# The Effect and Mechanism of Vascular Endothelial Growth Factor (VEGF) on Tumor Angiogenesis in Gallbladder Carcinoma

**Published:** 2019-04

**Authors:** Dongqing XU, Jianwen LI, Fangfang JIANG, Kaishuang CAI, Guangxue REN

**Affiliations:** 1. Department of Surgery (I), Yiling Hospital of Yichang, Yichang 443100, P.R. China; 2. Department of Surgery (II), Yiling Hospital of Yichang, Yichang 443100, P.R. China

**Keywords:** Vascular endothelial growth factor, Gallbladder carcinoma, Angiogenesis, GBC-SD cells, Apoptosis

## Abstract

**Background:**

To investigate the effect of vascular endothelial growth factor (VEGF) on tumor angiogenesis in gallbladder carcinoma.

**Methods:**

Fifty one patients with gallbladder carcinoma were enrolled as observation group. Thirty healthy people were included as control group. Chemically synthesized siRNA sequences targeting VEGF was transfected with VEGF-siRNA. A blank group (group B), a negative control group (transfected with independent sequence, group C), and an inhibition group (transfected with VEGF siRNA, group D) were established. Physiological saline was set as group A. The expression of VEGF was detected by qRT-PCR. The expression of VEGF protein was detected by Western blot. MVD was used to measure microvessel density. CCK-8, Transwell and flow cytometry were used to detect cell proliferation, invasion and apoptosis.

**Results:**

The tumor volume of nude mice and VEGF mRNA expression in group D was significantly smaller than that in group B and C (*P*<0.05). The MVD density in group B and C was significantly higher than that in group D (*P*<0.01). The proliferation of cells was detected from the 3rd day, and the proliferation of cells in the blank and negative control groups was faster than that of the inhibition group (*P*<0.05). The apoptosis rate of the blank group and the negative control group was lower than that of the inhibition group (*P*<0.001).

**Conclusion:**

VEGF is highly expressed in serum of patients with cholangiocarcinoma, it promotes angiogenesis, proliferation and invasion of gallbladder cancer cells, and inhibits apoptosis of tumor cells.

## Introduction

Carcinoma of gallbladder is one of the most common and most malignant tumors of the biliary system. Its incidence has increased from 7th to 5th in the bowel tumors at the end of the 20th century (1, 2). The incidence of gallbladder cancer has increased significantly, and it is on the rise (3). The early stage of the disease is very similar to the symptoms of cholecystitis, so early diagnosis is more difficult (4). This has seriously affected the quality of life of patients and caused a great economic and spiritual burden on the patient’s families (5).

Vascular endothelial growth factor (VEGF) is highly specific. VEGF has high expression in many kinds of tumors (6–8). Tumor angiogenesis is a very complicated process. Because the structure and function of neovascularization in tumor tissue are imperfect, leakage is easy to occur. Once there is leakage, there is a great threat to the patient’s life (9).

VEGF is expressed in gallbladder carcinoma but has not been studied further (10). Therefore, in this study, we conducted an evidence-based study on the role of VEGF in angiogenesis of gallbladder carcinoma and provided a more scientific basis for clinical treatment.

## Materials and Methods

### Clinical data of patients

Fifty one patients with gallbladder cancer diagnosed after surgery in Yiling Hospital of Yichang, Yichang, China were enrolled as observation group. There were 20 male patients and 31 female patients with an average age of 62.21±6.25 years. There were 44 patients with lymph node metastasis, 7 without lymph nodes metastasis, 40 patients with distant metastases, 11 patients without distant metastases. TNM staging: 9 patients with stage I+II, 42 patients with stage III+IV, 30 patients were poorly differentiated, and 21 patients were moderately and highly differentiated.

Thirty healthy people who underwent physical examination in our hospital without abnormal indicators were used as the control group. There was no statistically significant difference in gender and age between the two groups.

### Inclusion and exclusion criteria

Inclusion criteria: Patients and family members were informed and signed informed consent. All patients met the American Joint Committee on Cancer 8th Edition TNM classification, and patients were older than 18 years.

Exclusion criteria: Patients with other malignant tumors, patients undergoing radiotherapy and chemotherapy before admission, patients with severe heart, brain, lung, kidney, liver function defects.

### Animal sources

Sixteen BALB-C nude mice were purchased from Shanghai Slack Animal Laboratory, male, 4–5 weeks, weighing 25–30 g. They were randomly divided into groups A, B, C, and D, and each group had 4 nude mice. The mice were housed in separate cages at room temperature and had sufficient sunlight before being modeled. The noise was kept for less than 45 decibels and the mice were fed for one week.

### Construction of Gallbladder Carcinoma Nude Mice Model

Nude mice in group A were injected with equal doses of normal saline. Group B was injected with cells from the blank group. Group C was injected with cells from the negative control group. Group D was injected with cells from the inhibition group and injected every other day after each injection, a total of 3 injections. Then the tumor size was measured every 5 days (long axis L, short axis W, volume = 1/2*L* W2) to draw the growth curve. After 30 days, the nude mice were sacrificed, and tumors were taken from group B, C, and D for experiment. The gallbladder tissue of nude mice in group A was used for experiments. The rest was stored with liquid nitrogen. siRNA was constructed according to the literature of Elbashir et al (11) and synthesized by Shanghai Genepharma.

### Detection of VEGF Expression in Nude Mice Tissues and Cells by qRT-PCR

Total RNA was extracted using TRIzol reagent. cDNA extraction was performed according to the cDNA Synthesis Kit following the Kit instructions for reverse transcription. VEGF upstream primer: 5′-ATTTTTGTCTCATCCC-3′, downstream primer: 5′-GGTCACTACTTGCTCCTCGTCG-3′, RT-PCR kit was used to configure reaction system: 2.50 μL cDNA template, upstream and downstream primers 0.5 μL, 2.50 μL dNTPmix, 10*PCR buffer, 1.50 μL MgCl2, finally made up to 20 μL using ddH2O. The PCR conditions were as follows: predenaturation at 95°C for 5 minutes, 95 °C for 30 s, 65 °C for 45 s, and 72°C for 30 s for 40 cycles. U6 was used as the reference gene 5′-CTCGCTTCGGCAGCACA-3′5′-AACGCTTCACGAATTTGCGT-3. ‘. The resulting expression was calculated using the 2-ΔCt method.

### Western Blot Detection of VEGF Protein Expression in Nude Mice

Fifty mg gallbladder tissue was smashed, homogenized and digested with 1.5 mL 0.25 trypsin. The cells were lysed using RIPA lysate. Using 12% SDS-PAGE gel electrophoresis, the protein was electrotransfered to PVDF membrane. The membrane was placed in 5% skim milk blocked at 4°C overnight, washed with TBST, added nude mouse anti-human VEGF monoclonal antibody (1 : 400), incubated at room temperature for 2 h; washed again with TBST, added horseradish peroxidase-conjugated goat anti-rabbit secondary antibody, incubated for 1 h at room temperature, washed with TBST, and developed color. GAPDH was used as an internal control. Protein bands were used to measure the gray level of the protein bands using the Quantity one software. Product relative expression = target protein/internal control bands were grayscale.

### MVD determination

MVD was detected by CD34 immunohistochemical staining of the gallbladder tissue of two groups of nude mice by SABC method. The detection method was performed according to the manufacturer’s instructions (12). The MVD was counted, and a brown endothelial cell or cell groups distinct from the background was considered as a single microvessel. 5 slices were counted and the average was taking as the result.

### CCK-8 detection of cell proliferation

In the blank group, negative control group and inhibition group, 1*106 GBC-SD cells were transfected for 24 h, and the transfected GBCSD cells were then cultured in 96-well culture plates for 4 h at 6×103 /well. 10 μL of CCK-8 test solution was added daily. The plate reader was used to measure at 450 nm wavelength.

### Transwell detection of cell invasion

The transfected cells were selected to be made into a suspension and inoculated into the upper chamber, 4*104 cells per well, and cultured in RPMI-1640 medium (without FBS) for 24 h, and the RPMI-1640 medium was replaced (without FBS). Subsequently, 400 μL of 20% FBS RPMI-1640 medium was added to the lower chamber, and the cells were incubated for 36 h. A sterile cotton swab was used to wipe off the unpenetratized cells attached to the inner surface of the membrane and fixed with formaldehyde. Crystal violet (0.1%) staining was performed under a light microscope, and 6 replicate wells were set in the experiment and repeated 3 times.

### Flow Cytometry Apoptosis

The transfected cells was digested with 0.25% trypsin. The cell concentration was adjusted to 1*105/mL. Annexin V-FITC kit was used to perform count analysis on a flow cytometer (Beckman-CytoFLEX) and repeated 3 times.

### Elisa detection

Fifty μL of standard solution of different concentrations was separately added to the collected serum in the blank microwell. 50 μL of distilled water was added to the blank control well. Fifty μL of antibody was added; 40 μL of the sample was added to the remaining microwells followed by 10 μL of the biotinylated antibody. Subsequently, the plate was incubated at 37 °C for 30 min. Fifty μL of the enzyme standard solution was added to each well. Incubate at 37 °C for 60 min, wash the plate again for 5 times. Add horse-radish peroxidase labeled 100 μL/well sealing plate, incubate at 37 °C for 15 min in the dark. Color substrate TMB was added and the micro-plate reader was used for detection within 15 min to determine the maximum absorption wavelength of 450 nm. Set 3 sets of duplicate wells and repeat the experiment 3 times.

### Statistical analysis

We used the SPSS20.0 software package (Shanghai Cabit) for analysis, using GraphPad Prism 7 software for picture drawing; the enumeration data were expressed as rate (%) and used the chi-square analysis; measurement data were expressed as the mean ± standard deviation (Mean±SD) and used the *t*-test, and multiple groups were compared using analysis of variance. *P*<0.05 indicates that there is a statistically difference.

### Ethics aspects

This study was approved by the Ethics Committee of Yiling Hospital of Yichang. Patients and their families were informed in advance of the study, and singed an informed consent form.

## Results

### Serum VEGF expression in patients

The serum VEGF expression in the two groups was detected by ELISA method. The serum VEGF expression in the control group was 142.64±65.22 pg/mL, while the serum VEGF expression in the observation group was 288.41±86.25pg/mL. There was a significant difference in the expression of VEGF in serum (*P*<0.05). Further analysis of the relationship between VEGF and clinical data of the observation group found that VEGF showed significant differences in tumor differentiation, lymph node metastasis, distant metastasis and clinical stage (*P*<0.01) (Table 1).

**Table 1:** Relationship between VEGF and clinical data

***Factors***		***VEGF (pg/mL)***	***t***	***P***
Tumor differentiation				
	Low	352.54±49.54	7.736	<0.001
	Medium+High	244.68±48.22		
Lymph node metastasis				
	Yes	328.65±65.32	6.308	<0.001
	No	165.47±49.22		
Distant metastasis				
	Yes	388.41±52.21	6.921	<0.001
	No	256.21±69.22		
Clinical stage				
	I+II	175.65±40.65	6.667	<0.001
	III+IV	329.55±66.32		

### Tumor growth in nude mice in each group

**Fig. 1:**
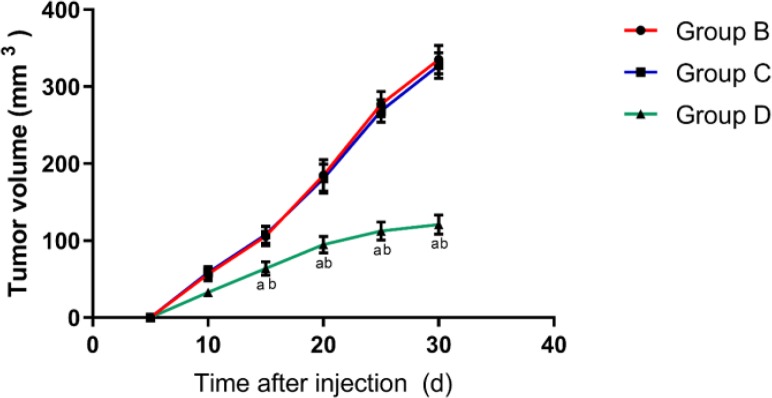
Comparison of tumor volume in nude mice in each group

We successfully constructed a gallbladder cancer tumor-bearing nude mouse model (the tumor tissues were identified for gallbladder cancer). During the modeling period, each group of nude mice grew well except one death in group B and group C. The tumor size of nude mice showed that group A had no tumors, and the tumor size of nude mice in group B and group C increased gradually with the increase of time, and there was no difference in tumor volume between the two groups. The tumor volume in group D also increased with time, but it was significantly smaller than that in group B and C (*P*<0.01) (Fig. 1).

### Expression of VEGF in Gallbladder Tissue and GBC-SD Cells

We detected the relative expression of VEGF mRNA in GBC-SD and gallbladder tissues of nude mice by real-time fluorescence quantitative PCR, and found that in the nude mouse model with successful modeling, and the relative expression of VEGF mRNA was statistically different among the groups of A, B, C, and D (F=8.731, *P*=0.024).

**Fig. 2:**
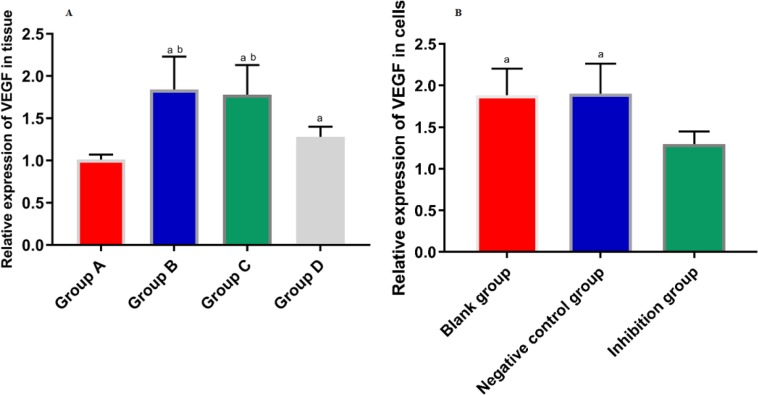
**A:** Expression of VEGF in tissues The expression of VEGF and VEGF mRNA in gallbladder tissues of nude mice in each group was different. The expression of VEGF mRNA in group A was lower than that in group B and C. There was no difference in the expression of VEGF mRNA between group B and group C. The expression of VEGF mRNA in group D was significantly lower than that of group B and C. Compared with group A, there was a significant increase in the expression of VEGF mRNA in group D. a indicates that there is a difference compared with the group B, and b indicates a difference compared with the group C **B:** Expression of VEGF in cells The expression of VEGF mRNA in each group was different. The relative expression of VEGF mRNA in the blank group and the negative control group was higher than that in the inhibition group, while there was no difference in the relative expression of VEGF mRNA between the blank group and the negative control group. a indicates that there is a difference compared with the inhibition group

The relative expression of VEGF mRNA in group A was significantly lower than that in group B and C (*P*_A VS B_=0.001, *P*_A VS C_=0.002). There was no significant difference in the relative expression of VEGF mRNA between the group B and C (*P*=0.186). The relative expression of VEGF mRNA in group D was significantly lower than that in group B and C (*P*_D VS B_=0.013, *P*_D VS C_=0.023). The relative expression of VEGF mRNA in group D was significantly higher than that in group A (*P*=0.047) (Fig. 2A). The relative expression of VEGF mRNA in each group of cells was also different (F=5.598, *P*=0.026). The relative expression of VEGF mRNA in the blank group and the negative control group was significantly higher than that in the inhibitory group (*P*_Blank group VS Inhibitory group_=0.019, *P*_Negative control group VS Inhibitory group_=0.016). There was no difference in the relative expression of VEGF mRNA between the blank group and the negative control group (*P*=0.921) (Fig. 2B).

### The relative expression of VEGF protein in nude mouse model

We detected the relative expression of VEGF protein in gallbladder tissue of nude mice by Western Blot and found that the relative protein expression of VEGF in each group of A, B, C, and D was statistically different (F=25.800, *P*=0.000). Among them, relative expression of VEGF protein in group A was significantly lower than that in group B and C (*P*_A VS B_<0.001, *P*_A VS C_<0.001).

In group A, there was no tumor in nude mice. The tumor volume of nude mice in group B and group C increased gradually with time, and there was no difference in tumor volume between the two groups. The tumor volume of group D also increased with time, but the tumor volume was less than in group B and C. a indicates that there is a difference compared with the group B, and b indicates a difference compared with the group C

**Fig. 3:**
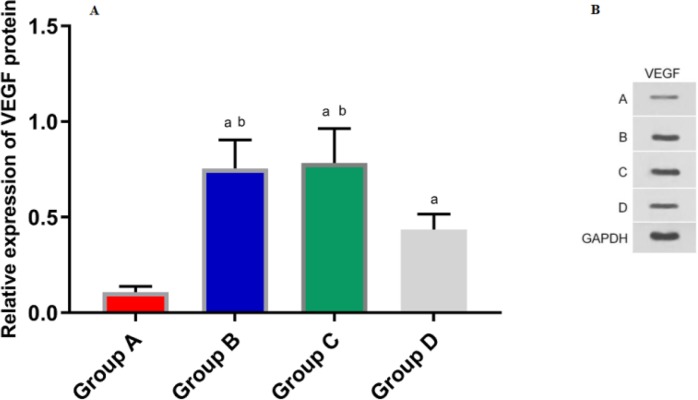
The expression of VEGF protein in gallbladder tissue of each group The relative expression of VEGF protein in gallbladder tissue of nude mice was detected by Western Blot. 3A: The expression of VEGF in each group was different. The relative expression of VEGF protein in group A was significantly lower than that of group B and C. There was no difference in the proportion of VEGF protein between group B and group C. The expression of VEGF in group D was significantly lower than that of group B and C. Compared with group A, the expression of VEGF increased in group D. a indicates that there is a difference compared with the A group, and b indicates a difference compared with the D group. 3B: Western Blotting protein map

There was no significant difference in the expression of VEGF protein between group B and group C (*P*=0.748). Relative expression of VEGF protein in group D was significantly lower than that in group B and C (P_D VS B_=0.004, P_D VS C_=0.002). The relative protein expression of VEGF in group D was significantly higher than that in group A (*P*=0.003) (Fig. 3).

### Nude mouse MVD situation

After staining, we used an optical microscope to count the MVD of gallbladder tissue in 4 groups of nude mice. The results showed that there was a statistically significant difference in MVD counts among the 4 groups (F=17.069, *P*=0.000). The sparse MVD of the brown cords in group A was significantly lower than that in the other three groups (*P*_A VS B_<0.001, *P*_A VS C_<0.001, *P*_A VS D_=0.036). The MVD density of cords in group B and C was significantly higher compared with that in group D (P_B VS D_=0.002, P_C VS D_=0.001). There was no difference in MVD between group B and group C (*P*=0.758) (Table 2).

**Table 2:** MVD situation of nude mice

***Group***	***MVD count (cord)***	***F***	***P***
A (n=4)	1.5±0.8		
B (n=4)	15.6±4.6^a^^b^		
C (n=4)	16.4±5.1^a^^b^	17.069	0.000
D (n=4)	5.5±1.9^a^		

Note:

a indicates a difference compared with group A (*P*<0.05),

b indicates a statistical difference with group D (*P*<0.05)

### Cell proliferation

**Fig. 4:**
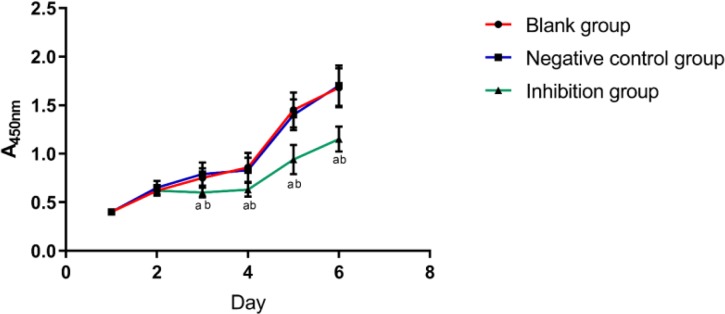
Growth of cells in each group We detected by CCK-8 method that the proliferation of cells in each group was different from the 3rd day. The proliferation of cells in the blank group and the negative control group was faster than that in the inhibition group, there was no difference in cell proliferation ability between the blank group and the negative control group a indicates a difference compared with the blank group, and b indicates a difference compared with the negative control group

We detected the proliferation of cells in each group by CCK-8 method and found that there was a difference in the proliferation of cells from the 3rd day (*P*<0.05). In the blank group and the negative control group, the proliferation rate of the cells was significantly faster than that of the inhibition group (*P*<0.05), and there was no difference in the cell proliferation ability between the blank group and the negative control group. (Fig. 4).

### Cell invasion

We detected the cells in each group by Transwell cell invasion assay and found that there were differences in cell invasion between the groups (F=19.509, *P*=0.001).

The number of permeated cells in the blank group and the negative control group was significantly higher than that in the inhibition group (*P*_Blank group VS Inhibitory group_<0.001, *P*_Negative control group VS Inhibitory group_=0.001), but there was no difference in the number of cell permeation between the blank group and the negative control group (*P*=0.505) (Table 3).

**Table 3:** Invasion of GBC-SD cells after transfection

***Group***	***Number of cell permeation***	***F***	***P***
Blank (n=4)	115.54±13.68		
		19.509	0.001
Negative control (n=4)	109.82±12.27^a^		
Inhibition (n=4)	68.38±8.35^a^		

Note:

a indicates a difference compared with the blank group (*P*<0.05)

### Apoptosis

We detected the apoptosis of cells in each group on the 6th day by flow cytometry and found that there was a difference in apoptosis between the groups (F=15.571, *P*=0.001). The apoptosis rate of the blank group and the negative control group was significantly lower than that of the inhibition group (*P*_Blank group VS Inhibitory group_=0.001, *P*_Negative control group VS Inhibitory group_=0.001). There was no difference in the apoptosis rate between the blank group and the negative control group (*P*=0.878).

## Discussion

Gallbladder cancer occurs mostly in the elderly over the age of 60, and the incidence of men is lower than that of women. Gallbladder carcinoma is characterized by high degree of malignancy, rapid infiltration and easy transfer, resulting in low surgical resection rate and multiple cleanings after resection (13). The occurrence and development of gallbladder cancer is regulated by many factors, among which apoptosis and proliferation disorders and tumor angiogenesis are closely related to its biological behavior (14, 15). Therefore, finding a way to prevent the occurrence, development and infiltration of gallbladder cancer has become the focus of attention today.

VEGF is the most effective pro-angiogenic factor nowadays, which binds to flt-1 and flt-1/KDR in a paracrine manner (16). The regulation of VEGF by miR-205 had a significant role in promoting the invasion of human ovarian cancer (17). Down regulation of VEGF had an inhibitory effect on lung cancer cell proliferation (18). However, there are few literatures related to the regulation of VEGF in gallbladder carcinoma. In this study, the expression of VEGF in the serum of the observation group was significantly higher than that of the control group, and the expression of VEGF was shown in another (19). MVD was significantly reduced in nude mice by inhibiting the expression of VEGF, indicating that VEGF expression is directly or indirectly associated with microvascular angiogenesis.

The cell proliferation ability of the blank group and the negative control group was significantly higher than that of the inhibition group. The number of cell permeation in the inhibition group was significantly lower than that in the other two groups. Finally, we found that inhibition of VEGF expression can effectively promote apoptosis. Inhibiting the expression of VEGF by targeting can inhibit the proliferation and migration of gastric cancer cells and promote the apoptosis of gastric cancer cells (20).

However, our current study still had some limitations. Firstly, this study did not detect VEGF expression in human tissues as an in vitro study, and the small number of our samples did not illustrate the accuracy of this study. Secondly, we did not dig deep into the mechanism of VEGF in gallbladder cancer. We hope to conduct clinical trials in future studies which will further verify the accuracy of our experiments.

## Conclusion

VEGF plays an important role in the promotion of angiogenesis in gallbladder carcinoma, promotes the proliferation, invasion, and apoptosis of tumor cells, and is expected to serve as a potential diagnostic indicator.

## Ethical considerations

Ethical issues (Including plagiarism, informed consent, misconduct, data fabrication and/or falsification, double publication and/or submission, redundancy, etc.) have been completely observed by the authors.
